# Adhesive Performance of Zirconia and Lithium Disilicate Maryland Cantilever Restorations on Prepared and Non-Prepared Abutment Teeth: An In Vitro Comparative Study

**DOI:** 10.3390/biomimetics10070413

**Published:** 2025-06-21

**Authors:** Tareq Hajaj, Ioana Elena Lile, Radu Marcel Negru, Serban Talpos Niculescu, Sami Stuparu, Mihai Rominu, Cosmin Sinescu, Paul Albu, Florina Titihazan, Ioana Veja

**Affiliations:** 1 Department of Prostheses Technology and Dental Materials, Faculty of Dentistry, “Victor Babes” University of Medicine and Pharmacy, 2 Eftimie Murgu Sq., 300041 Timisoara, Romania; tareq.hajaj@umft.ro (T.H.); samistuparu@gmail.com (S.S.); rominu.mihai@umft.ro (M.R.); sinescu.cosmin@umft.ro (C.S.); 2 Research Center in Dental Medicine Using Conventional and Alternative Technologies, Faculty of Dental Medicine, “Victor Babes” University of Medicine and Pharmacy, 9 Revolutiei 1989 Ave., 300070 Timisoara, Romania; radu.negru@upt.ro; 3 Department of Dental Medicine, Faculty of Dentistry, “Vasile Goldiș” Western University of Arad, 310025 Arad, Romania; lile.ioana@uvvg.ro (I.E.L.); veja.ioana@uvvg.ro (I.V.); 4 Department of Mechanics and Strength of Materials, Polytechnic University of Timișoara, 2 Piata Victoriei 2, 300006 Timisoara, Romania; 5 Department of Oral and Maxillofacial Surgery, Faculty of Dentistry, “Victor Babes” University of Medicine and Pharmacy, 2 Eftimie Murgu Sq., 300041 Timisoara, Romania; 6 Department of Pharmaceutical Sciences, Faculty of Pharmacy, “Vasile Goldiș” Western University of Arad, 86 L. Rebreanu St., 310414 Arad, Romania; albu.paul@uvvg.ro; 7 National Institute for Economic Research “Costin C. Kiritescu” of the Romanian Academy/Center for Mountain Economy, 49 Petreni St., 725700 Vatra Dornei, Romania

**Keywords:** adhesion, zirconia, ceramic, fixed partial denture, Maryland bridge, non-prepared, minimally invasive dentistry

## Abstract

Aim: This in vitro study aimed to evaluate the adhesive performance of zirconia and lithium disilicate Maryland cantilever restorations on prepared and non-prepared anterior abutment teeth. While conventional clinical protocols involve abutment tooth preparation, no-preparation (no-prep) restorations have emerged as a viable, minimally invasive alternative. This study compared the adhesion fracture resistance (N) of zirconia restorations on non-prepared enamel surfaces with those on prepared surfaces exposing the dentin. Additionally, the zirconia restorations were compared with lithium disilicate Maryland cantilever restorations, a more common yet costly alternative. Materials and Methods: Forty extracted anterior teeth were allocated into four groups based on preparation type (prepared vs. non-prepared) and material (zirconia vs. lithium disilicate). Each group received cantilevered single-unit FPDs fabricated via CAD/CAM and adhesively cemented using Variolink^®^ Esthetic DC. Standardized loading was applied using a universal testing machine, and the fracture resistance was recorded. Results: The fracture resistance ranged from 190 to 447 N in the zirconia groups and from 219 to 412 N in the lithium disilicate groups. When comparing all the zirconia versus all the lithium disilicate ceramic restorations, regardless of tooth preparation, no statistically significant difference was found (*p* = 0.752). However, the non-prepared restorations exhibited significantly higher fracture resistance than their prepared counterparts (*p* = 0.004 for zirconia; *p* = 0.012 for lithium disilicate ceramic). All the failures were attributed to tooth fracture, except one zirconia restoration, with no debonding observed. Conclusions: Both zirconia and lithium disilicate Maryland cantilever restorations demonstrated reliable adhesive performance when bonded using appropriate surface conditioning and adhesive protocols. Interestingly, the non-prepared designs exhibited higher fracture resistance than the prepared abutments, highlighting their potential advantage in minimally invasive restorative dentistry. Zirconia Maryland bridges, in particular, represent a cost-effective and mechanically resilient option for anterior single-tooth replacement.

## 1. Introduction

Tooth loss is a prevalent condition worldwide and can result from dental caries, periodontal disease, trauma, or congenital absence. Beyond esthetic and psychological consequences, missing teeth can impair mastication, speech, and occlusal balance [1,2,3]. Prosthodontics, a dental specialty focused on the restoration and replacement of teeth, offers several treatment options to rehabilitate partially edentulous patients, including removable dentures, fixed dental prostheses (bridges), and dental implants. Fixed partial dentures (FPDs), such as conventional multi-unit bridges or resin-bonded bridges, aim to restore function and esthetics while preserving oral structures [1,2]. Among these, minimally invasive options like cantilevered Maryland bridges have gained attention due to their conservative design and adhesive integration with the abutment tooth structure [4,5,6,7,8]. This study investigates the adhesive performance of such restorations, with a focus on zirconia and ceramic materials used in contemporary prosthodontic practice.

Tooth loss in the anterior region presents significant functional and esthetic challenges, often impairing mastication, speech, and a patient’s psychological well-being [1]. To rehabilitate a single missing front tooth, clinicians can consider dental implants, conventional fixed dental prostheses (FPDs), or removable partial dentures. However, implants may be contraindicated due to high cost, anatomical constraints, or patient preferences, and traditional FPDs typically require substantial removal of healthy structure from the adjacent teeth [1,2,9]. Removable prostheses are a non-invasive option but can be less comfortable and less esthetically pleasing than fixed solutions. Therefore, there is a strong clinical impetus to explore more-conservative treatment alternatives for anterior tooth replacement.

Resin-bonded fixed dental prostheses (RBFDPs), commonly known as Maryland bridges, have emerged as a conservative alternative in such cases [1]. First introduced over 40 years ago as the Rochette bridge, the Maryland bridge was designed to restore single anterior edentulous spaces while minimizing damage to the adjacent teeth by bonding a metal or ceramic wing to the enamel rather than preparing the abutment for a full-coverage crown [1,9]. As a result, this approach preserves most of the healthy tooth structure, reduces chairside time, and can achieve excellent esthetic outcomes—benefits that are especially important to young patients or when implant placement is not feasible [1,2,9]. Despite these advantages, Maryland bridges have not been universally adopted, largely due to concerns about long-term retention. Debonding has historically been a common mode of failure, and practitioners remain cautious about the durability of the resin–tooth interface—particularly when the prosthesis is made from zirconia, which requires specialized bonding protocols to achieve reliable adhesion [5,6,7].

In recent years, considerable improvements in bonding protocols have been made to enhance the longevity of resin-bonded bridges. In the case of zirconia frameworks, achieving a strong resin bond necessitates specific surface treatments. Airborne-particle abrasion of the zirconia (sandblasting with fine aluminum oxide particles) is performed to create micromechanical retention on the intaglio surface [3]. This is followed by the application of a phosphate monomer primer (10-methacryloyloxydecyl dihydrogen phosphate, 10-MDP), which chemically enhances the bond between the zirconia and the resin cement by forming P–O–Zr bonds through the interaction of the phosphate group with the zirconia surface, while the methacrylate group copolymerizes with the resin matrix [4,5]. Using a dual-cure resin cement (rather than a purely light-cured system) is recommended to ensure adequate polymerization through zirconia’s relatively opaque structure [6]. Furthermore, employing a meticulous total-etch, multi-step adhesive technique allows the clinician to maximize control over each bonding step, thereby improving the overall bond reliability [7].

Alongside zirconia, lithium disilicate glass-ceramic is another widely used material for minimally invasive fixed restorations. Lithium disilicate is favored for its superior esthetic qualities—such as enamel-like translucency—and its strong bonding capacity after hydrofluoric acid etching and silane treatment [1]. It also offers high fracture resistance (N) suitable for anterior use. Zirconia, by contrast, is an opaque high-strength ceramic that is less expensive [2] and has become increasingly attractive as a framework material thanks to the aforementioned advances in bonding. However, there is a knowledge gap regarding how these two materials compare in the context of Maryland bridges, especially when no tooth preparation is performed on the abutment. Most studies of resin-bonded bridges have focused on designs that include some form of tooth preparation or have examined traditional ceramic retainers, whereas fully “non-prepared” zirconia Maryland bridges are not yet well documented [8,9,10]. This lack of evidence may contribute to clinicians’ hesitation to use non-prepared designs in practice. Given the emphasis on conservative, metal-free restorations in modern dentistry, it is important to evaluate the performance of zirconia versus lithium disilicate cantilever bridges under both prepared and non-prepared conditions [11,12,13].

Therefore, the present in vitro study aims to evaluate the adhesive performance and fracture resistance of zirconia and lithium disilicate cantilever Maryland restorations bonded to anterior abutment teeth, both with and without tooth preparation. By investigating the combined influence of material type and preparation approach, this study seeks to provide clinically relevant data to support evidence-based decision-making in minimally invasive restorative dentistry [3,5,9,14].

From a biomimetic perspective, this study investigates how ceramic materials with distinct microstructural properties—such as zirconia’s high fracture toughness and lithium disilicate’s enamel-like translucency—interact with biological substrates under adhesive conditions. The adhesive strategies employed aim to replicate the hierarchical bonding mechanisms observed in natural dentin–enamel interfaces, using surface conditioning and functional monomers to establish durable micromechanical and chemical coupling. The comparison between prepared and non-prepared abutments further reflects a bioinspired approach, seeking to minimize structural loss while optimizing interfacial performance between synthetic ceramics and the native tooth structure.

## 2. Materials and Methods

This in vitro study was approved by the Ethics Committee of the “Victor Babes” University of Medicine and Pharmacy, Timisoara (Protocol No. 89/01.10.2022 rev. 2024). Forty anterior teeth (incisors and canines), extracted for orthodontic or periodontal reasons, were collected after obtaining written informed consent. The teeth were stored in saline solution until use.

### 2.1. Inclusion and Group Allocation

The forty selected teeth were randomly assigned to four groups (n = 10) using a computer-generated randomization sequence to minimize allocation bias. Only teeth with intact palatal and proximal surfaces, free from caries, restorations, or demineralization, were included. Teeth with endodontic treatment, loss of hard tissue, or composite fillings were excluded. The specimens were grouped based on restorative material (zirconia or lithium disilicate) and abutment condition (prepared or non-prepared).

Group 1: Zirconia restorations on non-prepared teeth.
Group 2: Zirconia restorations on prepared teeth.
Group 3: Lithium disilicate restorations on non-prepared teeth.
Group 4: Lithium disilicate restorations on prepared teeth.

For the prepared groups (Groups 2 and 4), standardized abutment preparation was performed by an experienced prosthodontist (Author M.R.) using ×3.5 magnification and high-speed rotary instrumentation under constant water spray. Fine-grit chamfer diamond burs (Komet 847KR 016, Lemgo, Germany) were used to selectively reduce the palatal surface and one proximal surface of each abutment tooth to a depth of approximately 0.5 mm, following the enamel contour to preserve structure and avoid unnecessary dentin exposure. The preparation margins were chamfered and polished to optimize adhesive adaptation. In the non-prepared groups (Groups 1 and 3), no surface reduction was performed; the restorations were adapted directly to the unaltered enamel, with a minimum material thickness of 0.5 mm.

All the restorations maintained a connector surface of at least 7 mm^2^, according to the manufacturer’s recommendations. Following preparation (if applicable), all the teeth were embedded in acrylic resin up to the cement–enamel junction (CEJ) to simulate clinical conditions and provide mechanical stability during testing.

**Figure 1:**
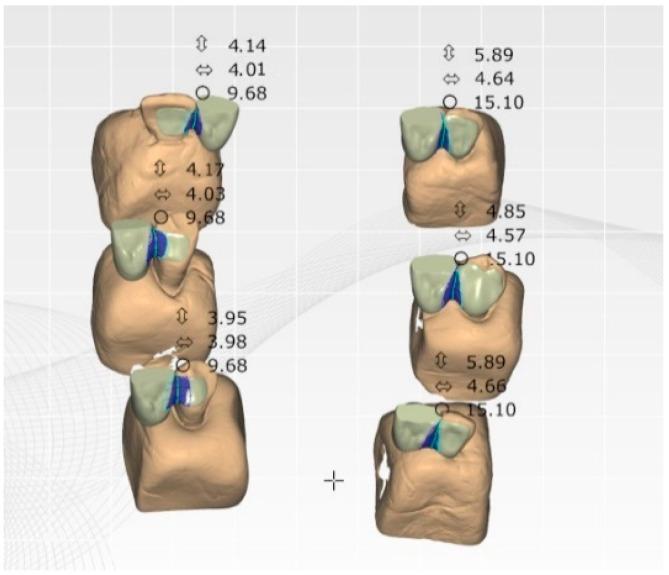
Schematic of the cantilever Maryland bridge design with a single proximal wing.

The final design used in all the groups was a single-retainer cantilever Maryland bridge with one proximal wing (Figure 1).

### 2.2. Restoration Design and Fabrication

All the restorations were designed as single-retainer cantilever Maryland bridges (one-wing design). The connector area was standardized to ≥7 mm^2^, as per the manufacturer’s recommendations. Digital impressions were obtained using intraoral scanning, and the restorations were fabricated via CAD/CAM.

Zirconia material: DD Bio ZWISO 3Y-TZP (Dental Direkt GmbH, Spenge, Germany).

Ceramic (lithium disilicate) material: IPS e.max^®^ Press MO 0 (Ivoclar Vivadent, Schaan, Liechtenstein).

Each restoration was adapted according to the specific tooth preparation and trial-fitted prior to cementation.

### 2.3. Surface Conditioning Protocols

The zirconia restorations were sandblasted (50 µm Al_2_O_3_, 2.0 bar, 10–15 s at 2–2.5 cm distance). After rinsing and drying, Z-Prime™ Plus (Bisco, Schaumburg, IL, USA) was applied to the bonding surface and air-dried for 2–3 s [11,15].

The lithium disilicate restorations were etched with 9.5% hydrofluoric acid (20 s), rinsed, and dried. Silane was applied as per the manufacturer’s instructions.

**Figure 2:**
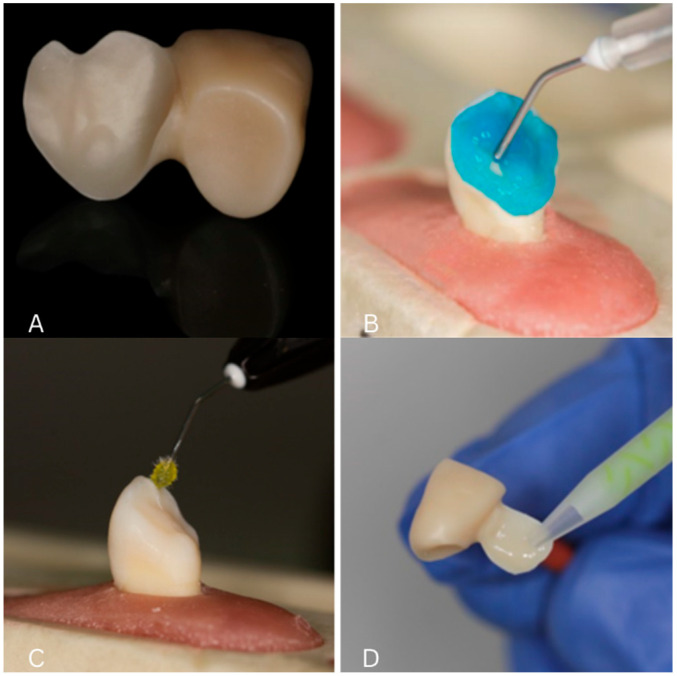
(**A**) Final restoration after sandblasting; (**B**) etching of lithium disilicate restorations with 9.5% hydrofluoric acid; (**C**) adhesive application; (**D**) application of dual-cure resin cement to the intaglio of the restoration.

Before bonding, all the teeth were professionally cleaned using a toothbrush and fluoride-free prophylactic paste to remove any surface debris and avoid interference with the adhesive process. The enamel and dentin were sandblasted (50 µm Al_2_O_3_, 2.0 bar), followed by phosphoric acid etching: 30 s for enamel and 15 s for dentin. After rinsing and gentle drying, a universal bonding agent (Adhese^®^ Universal, Ivoclar Vivadent, Schaan, Liechtenstein) was applied followed by final light curing for 20 s per surface using a LED polymerization light (Bluephase N^®^, Ivoclar Vivadent; 800 mW/cm^2^) (Figure 2).

### 2.4. Cementation Procedure

All the restorations were adhesively luted using Variolink^®^ Esthetic DC dual-cure resin cement (Ivoclar Vivadent). The cement was applied in a single layer to the restoration, which was seated under slight pressure. Polymerization was initiated from the buccal side to direct shrinkage toward the tooth. Glycerin gel was applied to the margins to eliminate the oxygen-inhibited layer during final curing. The cement was allowed to self-cure simultaneously.

### 2.5. Mechanical Testing

Specimens were embedded in acrylic up to the cement–enamel junction to simulate periodontal support. Load testing was performed using a Zwick/Roell ProLine Z005 universal testing machine (Zwick Roell GmbH, Ulm, Germany) under displacement control (1 mm/min). A custom-made zirconia rod applied a perpendicular force at 3 mm from the palatal wing–cantilever junction.

**Figure 3:**
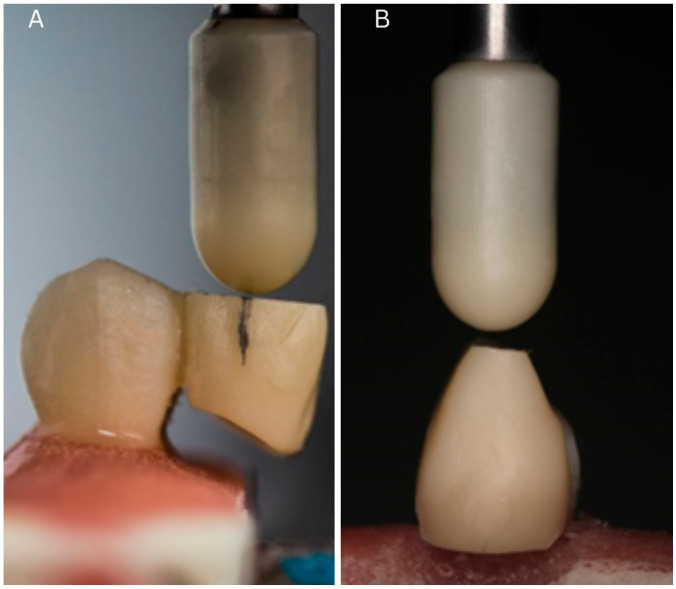
Experimental procedure: (**A**) load application during mechanical testing (3 mm from the palatal wing); (**B**) custom-made zirconia rod used for force delivery.

Failure was defined as a fracture of the tooth or restoration, or visible debonding. The fracture resistance was recorded continuously, and the peak failure load (in N) was documented for each specimen (Figure 3).

### 2.6. Statistical Analysis

The required sample size was calculated prior to testing based on data from similar in vitro studies evaluating fracture resistance in bonded restorations. A minimum of 7 specimens per group was estimated to achieve a power of 80% at a significance level of α = 0.05. To increase robustness, 10 samples per group were included. The independent variables in the study were as follows: restorative material: zirconia vs. lithium disilicate; abutment preparation: prepared vs. non-prepared (no-prep). The dependent variables were as follows: fracture resistance (expressed in Newtons); mode of failure (adhesive, cohesive, or mixed).

The data normality was assessed using the Chi-square test. The mean fracture resistance values were compared using two-sample *t*-tests, and Levene’s test was applied to assess the equality of variances. Statistical analysis was performed using MedCalc^®^ version 23.0.6 (MedCalc Software Ltd., Ostend, Belgium), with a significance level of *p* < 0.05.

## 3. Results

**Figure 4:**
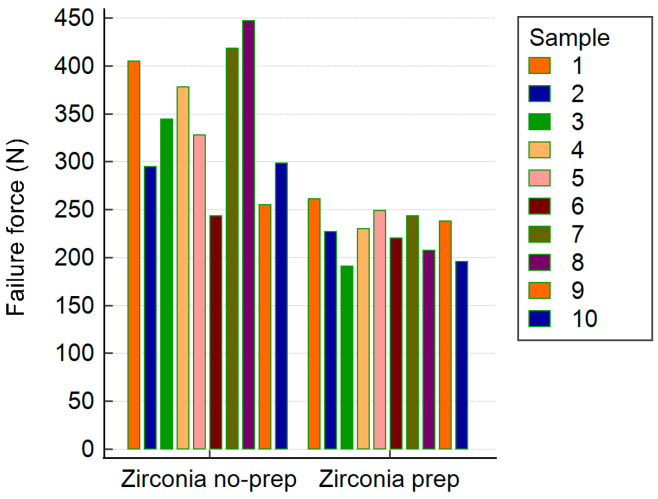
Recorded failure values for all zirconia samples.

The zirconia restorations showed fracture resistance ranging from 191 to 447 N. The non-prepared group had a significantly higher mean fracture resistance (M = 341.19 N, SD = 69.71) than the prepared group (M = 226.19 N, SD = 22.88). This difference was statistically significant (*t*(10.92) = 4.96, *p* < 0.001, 95% CI [63.88, 166.12]). Levene’s test indicated unequal variances (*p* = 0.004). Most failures involved tooth fracture; only one zirconia restoration fractured, and no debonding was observed (Table 1, Figure 4).

**Figure 5:**
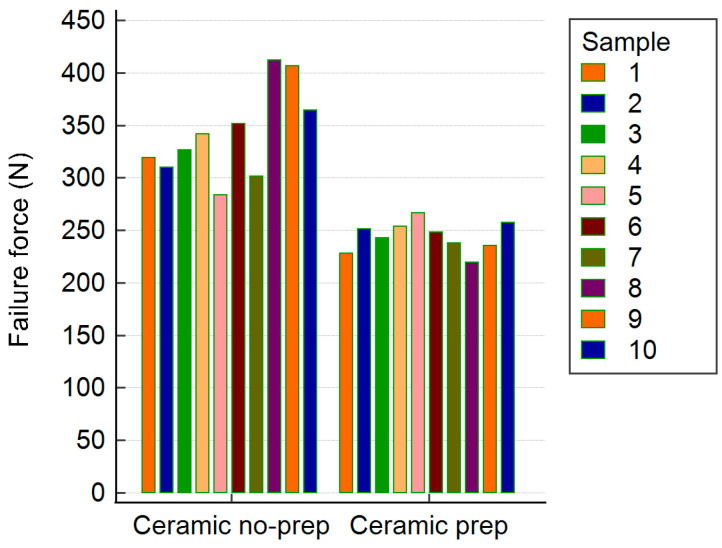
Recorded failure values for all lithium disilicate samples.

The lithium disilicate restorations showed similar trends, with a fracture resistance between 219 and 412 N. The non-prepared lithium disilicate group had a significantly higher mean resistance (M = 341.71 N, SD = 42.89) than the prep group (M = 243.94 N, SD = 14.21), with *t*(10.95) = −6.84, *p* < 0.001, and 95% CI [−129.24, −66.30]. Levene’s test confirmed unequal variances (*p* = 0.012). All the failures were due to tooth fracture, with no debonding in any lithium disilicate sample (Table 2, Figure 5).

When comparing zirconia with lithium disilicate restorations, regardless of preparation, no statistically significant difference was observed (*p* = 0.752) (Table 3). The zirconia restoration group had lower values than the ceramic restoration group, but the difference was not statistically significant: a *p*-value of 0.752 was obtained, which is above the specified significance level of 0.05 (Table 3).

All the restorations survived cementation and underwent mechanical testing. The fracture resistance values ranged from 190.72 N to 447.00 N across groups.

### 3.1. Group Comparisons

**Figure 6:**
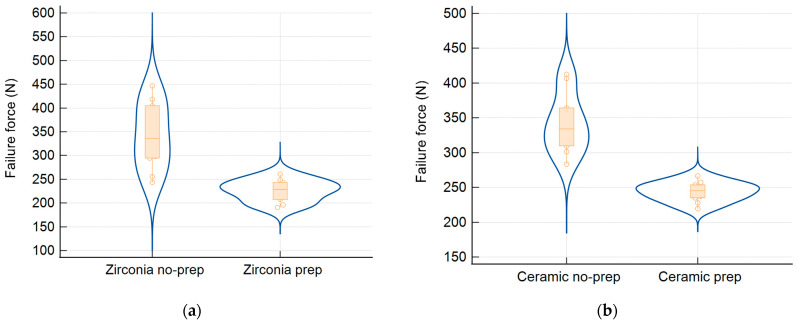
Violin plots show the distributions of fracture resistance for (**a**) zirconia and (**b**) ceramic restorations involving non-prepared and prepared abutment teeth.

Zirconia Groups (Figure 6a and Table 1):

Non-prepared zirconia restorations showed a significantly higher mean fracture resistance (341.19 ± 69.71 N) than prepared ones (226.19 ± 22.88 N; *p* < 0.001).

All the failures were due to tooth fracture, except one case of zirconia restoration fracture. No debonding occurred.

Lithium Disilicate Groups (Figure 6b and Table 2):

Non-prepared lithium disilicate restorations exhibited a higher fracture resistance (341.71 ± 42.89 N) than the prepared group (243.94 ± 14.21 N; *p* < 0.001).

All the failures involved tooth fractures; no debonding was observed.

Material Comparison (All Conditions Combined):

No significant difference was found between zirconia and lithium disilicate restorations when averaged across preparation types (*p* = 0.752) (Table 4).

### 3.2. Failure Mode

In all the groups, the dominant failure mode was tooth fracture. No adhesive failures (debonding) were recorded, supporting the effectiveness of the bonding protocols used.

### 3.3. Summary of Fracture Resistance Values (Table 4)

Violin plots of the fracture resistance distribution are presented in Figure 6, illustrating the data spread and median values for all four groups.

## 4. Discussion

This study supports the clinical viability of zirconia and lithium disilicate Maryland cantilever restorations as minimally invasive alternatives for anterior tooth replacement. When appropriate bonding protocols are followed, both materials demonstrate high adhesive strength and fracture resistance exceeding the average anterior bite force (100–150 N), without any occurrence of debonding. Clinical studies confirm these findings: Yazigi et al. [14] reported favorable outcomes using cantilevered single-retainer all-ceramic zirconia restorations in anterior and posterior cases, especially in patients with contraindications to implants. Similarly, Shah et al. [1] and systematic reviews [16] highlight the success of all-ceramic resin-bonded prostheses, with reported survival rates between 76% and 100%. Despite these advantages—including reduced invasiveness, excellent esthetics, and preservation of tooth structure—Maryland bridges remain underutilized, largely due to historical concerns about debonding and technique sensitivity. Increasing clinician familiarity with modern adhesive techniques and material-specific protocols could facilitate broader adoption of these restorations, particularly in young patients or those for whom implant therapy is unsuitable [1,9,14].

Although statistically significant differences in fracture resistance were observed between prepared and non-prepared abutment teeth (*p* < 0.001), the clinical relevance of these differences must be contextualized. The lowest recorded fracture resistance value in this study (190.72 N) still exceeds the average bite force in the anterior region (100–150 N) [12,13], indicating that all the restorations tested may perform adequately under physiological conditions. However, the higher values observed in the non-prepared groups could offer additional safety margins in patients with parafunctional habits or increased occlusal demands. This distinction has been added to provide a more balanced clinical interpretation of the findings.

An important observation is that all the failures were due to tooth fracture, with no adhesive failures noted. This supports the strength of the adhesive protocols used. Notably, one zirconia restoration itself fractured. While no fractographic evaluation was performed, possible explanations include internal flaws within the zirconia structure, stress concentrations at the connector site, or localized surface defects introduced during sandblasting. This isolated case highlights the need for further studies with microscopic failure analysis to better understand the mechanical behavior of such restorations.

Although no statistically significant difference was found between zirconia and lithium disilicate restorations overall (*p* = 0.752), zirconia exhibited the highest individual fracture resistance value (447 N) and had a broader range. This reflects zirconia’s superior mechanical properties, including higher fracture toughness and resistance to crack propagation, which may provide advantages in high-stress clinical situations. While the present discussion has primarily focused on bonding protocols, future investigations should also take into account the intrinsic mechanical characteristics of the materials, which may influence long-term performance and restoration selection.

Adhesive bonding to both zirconia and lithium disilicate plays a critical role in the long-term success of resin-bonded restorations. Lithium disilicate achieves a high bond strength through hydrofluoric acid (HF) etching, which creates micromechanical retention, followed by silane application, which enables chemical bonding with the resin matrix [16,17,18]. In contrast, zirconia is chemically inert to HF and relies on mechanical roughening and the use of phosphate monomers such as 10-MDP, which form stable chemical bonds (P–O–Zr) between zirconia oxide and the functional monomer, while the methacrylate group copolymerizes with the resin cement [19,20]. In our study, the absence of cement residue on zirconia surfaces post-fracture, compared with its presence on enamel, supports this mechanism. Universal adhesive systems that combine both silane and 10-MDP have shown promising results, simplifying clinical protocols while maintaining or enhancing bond strength [21,22]. Moreover, properly conditioned zirconia surfaces, when bonded with MDP-containing cements, demonstrate long-term durability in vitro and in vivo [23,24], and recent studies suggest that the influence of surface treatment and adhesive strategy often outweighs the impact of the specific resin cement used [25,26,27,28,29,30].

Longitudinal in vivo studies are essential to determine survival rates; the risk of debonding; marginal integrity; and patient-reported outcomes, such as satisfaction, functionality, and esthetics [31,32,33]. Incorporating artificial aging protocols can help simulate oral conditions more realistically and predict long-term adhesive performance [34]. Comparative studies utilizing a wider array of bonding agents, alternative surface treatments (e.g., tribochemical silica coating, laser irradiation), and modified abutment geometries are also warranted to broaden clinical applicability [35]. Moreover, the integration of fully digital workflows, including CAD/CAM design and fabrication, as well as the exploration of bioactive or smart adhesive materials, may pave the way for optimizing minimally invasive restorative strategies in fixed prosthodontics [36].

Tooth loss is a prevalent condition worldwide and can result from dental caries, periodontal disease, trauma, or congenital absence. Beyond esthetic and psychological consequences, missing teeth can impair mastication, speech, and occlusal balance [13]. Prosthodontics, a dental specialty focused on the restoration and replacement of teeth, offers several treatment options to rehabilitate partially edentulous patients, including removable dentures, fixed dental prostheses (bridges), and dental implants. Fixed partial dentures (FPDs), such as conventional multi-unit bridges or resin-bonded bridges, aim to restore function and esthetics while preserving oral structures [1,2]. Among these, minimally invasive options like cantilevered Maryland bridges have gained attention due to their conservative design and adhesive integration with the abutment tooth structure [1,9,14]. This study investigates the adhesive performance of such restorations, with a focus on zirconia and ceramic materials used in contemporary prosthodontic practice [3,5,6,37].

Recent interdisciplinary research has emphasized the importance of material innovation and non-invasive diagnostic methods in enhancing the performance of dental restorations. Magnetodielectric studies on polymer-based composite systems have provided valuable insights into the behavior of smart materials under external stimuli relevant to dental applications [38]. Optical coherence tomography has been effectively employed to monitor structural changes in metal–ceramic prostheses during thermal processing, demonstrating the potential of non-destructive evaluation for quality control [39]. Investigations on the reinforcement of adhesive interfaces with magnetic nanoparticles revealed improvements in mechanical stability and bonding performance, assessed through advanced imaging techniques such as micro-CT and optical microscopy [40].

This study has several limitations. First, the use of different tooth types (central incisors, lateral incisors, and canines) introduced variability in the available bonding surface, potentially influencing adhesion and fracture behavior. Canines typically offer a larger palatal area and thicker enamel, which may provide more-favorable bonding conditions compared with incisors. Future studies should standardize tooth selection to either all incisors or all canines to minimize this confounding factor. Additionally, this was an in vitro study, and the results may not fully replicate intraoral conditions such as humidity, temperature variation, and dynamic occlusal forces.

Future studies should incorporate artificial aging methods (e.g., thermocycling, cyclic fatigue) to simulate clinical conditions more closely [12]. Long-term clinical trials comparing zirconia and ceramic Maryland bridges under varying loads and anatomical locations are needed [13]. Investigating patient-reported outcomes—such as comfort, esthetics, and satisfaction—will also provide important clinical insight.

Additional research should evaluate new adhesive materials and methods, including bioactive cements and smart adhesives. Comparative studies of bonding agents, surface treatments (e.g., tribochemical silica coating, laser conditioning), and abutment geometries are warranted. Finally, the integration of digital workflows (CAD/CAM fabrication, intraoral scanning) and advanced diagnostics such as OCT and micro-CT could enhance the precision and long-term success of adhesive restorations.

## 5. Conclusions

Within the limitations of this in vitro study, both zirconia and lithium disilicate Maryland cantilever restorations demonstrated reliable adhesive performance when bonded using appropriate protocols. Non-prepared designs showed significantly higher fracture resistance than prepared teeth, regardless of material, suggesting a clinical advantage for conservative treatment. Zirconia restorations, in particular, offer a durable and cost-effective solution for anterior single-tooth replacement when esthetics and tissue preservation are priorities.

## Figures and Tables

**Table 1:** Mean failure value of zirconia samples.

Zirconia Samples	Max Fracture Resistance (N) Non-Prepared Teeth	Max Fracture Resistance (N) Prepared Teeth
1	404.86	260.81
2	294.71	227.01
3	344.3	190.72
4	378.03	230.03
5	327.84	248.58
6	243.37	220.33
7	418.42	243.3
8	447	207.45
9	255.17	237.84
10	298.16	195.81
Mean Values	341.19	226.19
Median	336.07	228.52
SD	69.71	22.88

**Table 2:** Mean failure value for lithium disilicate restoration samples.

Lithium Disilicate Restorations	Max Fracture Resistance (N) Non-Prepared Teeth	Max Fracture Resistance (N) Prepared Teeth
1	319.41	227.94
2	309.74	251.08
3	326.32	242.59
4	342.02	253.62
5	283.41	266.44
6	351.57	248.35
7	301.55	237.69
8	412.28	219.45
9	406.55	235.14
10	364.29	257.11
Mean Values	341.71	243.94
Median	334.17	245.47
SD	42.89	14.21

**Table 3:** Failure values of zirconia and lithium disilicate restorations.

	Zirconia Restorations	Lithium Disilicate Restorations
Mean	283.69	292.83
SD	77.65	59.02
Minimum	190.72	219.45
Maximum	447	412.28
95% CI	247.34–320.03	265.21–320.45

SD—Std. deviation; CI—confidence interval for mean.

**Table 4:** Summary of fracture resistance values per group.

Group	Material	Preparation	Mean ± SD (N)	Min–Max (N)
Group 1—Zirconia, Non-prepared	Zirconia	No	341.19 ± 69.71	243.37–447.00
Group 2—Zirconia, Prepared	Zirconia	Yes	226.19 ± 22.88	190.72–260.81
Group 3—Lithium Disilicate, Non-prep	Lithium Disilicate	No	341.71 ± 42.89	283.41–412.28
Group 4—Lithium Disilicate, Prepared	Lithium Disilicate	Yes	243.94 ± 14.21	219.45–266.44

SD—Std. deviation.

## Data Availability

The data will be available from the corresponding authors upon reasonable request.
